# The effect of rare variants on inflation of the test statistics in case–control analyses

**DOI:** 10.1186/s12859-015-0496-1

**Published:** 2015-02-20

**Authors:** Ailith Pirie, Angela Wood, Michael Lush, Jonathan Tyrer, Paul DP Pharoah

**Affiliations:** 1 Department of Public Health and Primary Care, Strangeways Research Laboratory, University of Cambridge, 2 Worts’ Causeway, Cambridge, CB1 8RN UK; 2 Department of Oncology, Strangeways Research Laboratory, University of Cambridge, 2 Worts’ Causeway, Cambridge, CB1 8RN UK

**Keywords:** Rare variants, Inflation, Genetic association, Case–control analyses, Likelihood ratio test, Score test, Wald test, Population structure

## Abstract

**Background:**

The detection of bias due to cryptic population structure is an important step in the evaluation of findings of genetic association studies. The standard method of measuring this bias in a genetic association study is to compare the observed median association test statistic to the expected median test statistic. This ratio is inflated in the presence of cryptic population structure. However, inflation may also be caused by the properties of the association test itself particularly in the analysis of rare variants. We compared the properties of the three most commonly used association tests: the likelihood ratio test, the Wald test and the score test when testing rare variants for association using simulated data.

**Results:**

We found evidence of inflation in the median test statistics of the likelihood ratio and score tests for tests of variants with less than 20 heterozygotes across the sample, regardless of the total sample size. The test statistics for the Wald test were under-inflated at the median for variants below the same minor allele frequency.

**Conclusions:**

In a genetic association study, if a substantial proportion of the genetic variants tested have rare minor allele frequencies, the properties of the association test may mask the presence or absence of bias due to population structure. The use of either the likelihood ratio test or the score test is likely to lead to inflation in the median test statistic in the absence of population structure. In contrast, the use of the Wald test is likely to result in under-inflation of the median test statistic which may mask the presence of population structure.

**Electronic supplementary material:**

The online version of this article (doi:10.1186/s12859-015-0496-1) contains supplementary material, which is available to authorized users.

## Background

Population stratification – allele frequency differences between cases and controls due to systematic ancestry differences – can cause spurious results in genetic association studies [1-4]. The bias associated with population stratification can be reduced by ensuring cases are matched to controls based on self-reported ethnicity or ancestry [5]. However, self-reported ancestry is not a perfect substitute for genetic ancestry [6]. In addition, unlinked markers that have differing frequencies between populations can then be used to estimate the ancestry of sampled individuals and this information can then be used to adjust for ancestry when testing for associations within subpopulations [7]. Nevertheless, the detection of bias due to cryptic population sub-structure is an important step in the evaluation of the findings of genetic association studies. The standard approach for detecting bias in an analysis of a large number of genetic variants is to test for inflation of the test statistics by calculating the ratio of the observed test statistic with the expected test statistic at a given quantile, typically the median [8]. The effect of population structure in the analysis of rare variants and in particular in the use of gene-based tests on rare variants has been widely studied [9-14]. Mathieson et al. [14], show that population structure in rare variants leads to increased levels of inflation in the test statistic in comparison to that observed in tests of common variants. In addition, inflation can still be observed when there is low levels of population structure in common variants due to differing population structure across variant frequencies [14]. However, over-dispersion of the test statistic may occur in the absence of population structure and may occur as a result of the properties of the test itself. The analyses presented in this paper were motivated by the observation of substantial inflation in the test statistics related to rare variant association testing in a case–control analysis using logistic regression. In contrast, inflation was minimal in an analysis of common variants for the same sets of samples. There has been extensive evaluation of the properties of the likelihood ratio test, the Wald test, and the score test in case–control analyses with respect to Type 1 error rates. These have focussed on test performance at the extremes of the distribution [15,16]. For example, Xing et al. recently reported type 1 error rates for these three tests in a case–control genetic association analysis investigating low-frequency variants [16]. The likelihood ratio test maintained controlled type 1 error rates whereas the Wald test and the score test were conservative particularly at the extreme upper tail of the distribution. However, there has been less reported research on the properties of these tests at the lower centiles of the distribution relevant for the estimation of over-dispersion. We therefore sought to evaluate the properties of the three most commonly used tests, the likelihood ratio test, the Wald test, and the score test, when testing rare variants for association.

## Methods

Our analysis was split into two parts. Firstly, we investigated how rare a variant had to be before its frequency had an effect on the inflation in the test statistic under each of the three models in a case–control analysis. Secondly, we investigated the size of the effect on inflation that could be expected in a rare variant analysis. We simulated datasets with a distribution of minor allele frequencies typical of a rare variant analysis and calculated the inflation for each of the three tests.

Throughout all analyses, association with risk of a phenotype was tested using logistic regression. The likelihood ratio test, score test and Wald test were used to generate test statistics for each variant. The test statistics for all three tests should follow an asymptotic distribution of χ^2^ with 1 degree of freedom. R code used to program the simulations can be found in Additional file 1.

### Calculating the inflation

A reportedly robust [8] measure of inflation in genetic association studies is the ratio of the observed median χ^2^ statistic over the expected median of the χ^2^ distribution under the null hypothesis of no association. Therefore, we calculated the inflation factor (λ) for each test based on the median of the test statistics for each dataset. For example, the inflation factor of the likelihood ratio test on variants with 10 heterozygotes is equal to the median of all the 100,000 likelihood ratio test statistics on variants with 10 heterozygotes, divided by 0.456, the median value of the χ^2^ distribution on 1 degree of freedom.

1 $$ \lambda =\frac{\mathrm{median}\left({T}_1,{T}_2,\dots {T}_N\right)}{0.456} $$

where T_i_ = the association test statistic for the i_th_ variant in *N*. An alternative to the ratio of the observed to expected median test statistic is the observed to expected mean test statistic. However, the mean test statistic is less robust to the effect of outliers [17].

### Determining the frequency threshold

In order to investigate the frequency threshold below which rare variants have an influence on inflation, we simulated variants with between 1 and 50 heterozygotes. We generated a dataset for each variant frequency comprising genotypes for 100,000 variants under the assumptions of no disease-risk and no population structure. For each dataset we defined a list of genotypes with length equal to the total number of cases and controls. The genotypes were either major allele homozygotes (0) or heterozygotes (1) as minor allele homozygotes were considered too rare to be included. The number of heterozygotes in the list was equal to the variant frequency. Then genotypes for each individual were selected randomly without replacement from the list of genotypes. The assignment of genotypes was repeated 100,000 times to complete the dataset. Phenotypic indicators (1 for a case, 0 for a control) were assigned in equal proportions and at random to individuals.

Each of these datasets were then tested, using each of the three tests, for association with risk of a phenotype and the inflation factor (λ) for each test was calculated. The level of inflation was then compared between the three tests for each of the variant frequencies. This analysis was conducted across 5,000 and 10,000 samples in order to investigate whether any effect on inflation was related to either the number or the proportion of rare allele carriers (heterozygotes) in the sample.

### Determining the size of the effect of frequency on inflation factor (λ)

We aimed to estimate the size of the effect that low variant frequency has on the level of inflation in the test statistics from the likelihood ratio test, the Wald test and the score test when testing for association in a case–control analysis. To do this we generated a genotypic dataset (dataset 1) over 7,047 samples assuming the null hypothesis, with a set of variants with a minor allele frequency distribution equivalent to that of the variants on the Illumina HumanExome Beadchip. This genotyping array is designed for the analysis of rare coding variants and has a minor allele frequency distribution typical of rare variant studies. We used a sample size of 7,047, equal to the sample size of the Illumina HumanExome Beadchip array from an ovarian cancer case–control study. Variants with fewer than 10 heterozygotes were generated in the same way as above whereas variants with greater than 10 heterozygotes were generated from a multinomial distribution with genotype probabilities following Hardy Weinberg Equilibrium.

We then simulated 1,000 datasets of 100,000 variants over 7,047 samples. The datasets of 100,000 variants were composed of 63,229 variants selected with replacement from the variants below the frequency threshold in dataset 1 and 36,771 variants selected with replacement from the variants with minor allele frequencies above the threshold in dataset 1. This ratio is comparable to the ratio of variants below and above the frequency threshold in the HumanExome Beadchip array. Each of the 1,000 datasets were tested for association with risk using the same phenotype dataset which was generated by assigning case and control status to individuals evenly and at random. The inflation factor (λ) was then calculated for each of the three tests using the median and mean test statistics. The test statistics for variants with minor allele frequencies above and below the frequency threshold were then considered separately and inflation factors (λ) were calculated separately for each set using the median and mean test statistics. The levels of inflation for each test were then averaged across the 1,000 datasets and the variability was estimated using the standard error.

## Results and discussion

### Determining the frequency threshold

**Figure 1:**
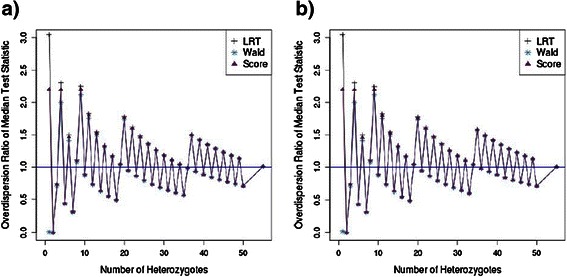
**The level of inflation of the median test statistic for variants, with increasing numbers of heterozygotes, in a case–control analysis using the likelihood ratio test, the Wald test and the score test. a)** The over-dispersion ratio at the median test statistic in a case–control analysis for a sample size of 5,000 with up to 50 heterozygotes. **b)** The over-dispersion ratio at the median test statistic in a case–control analysis for a sample size of 10,000 with up to 50 heterozygotes.

The inflation for the three test statistics in the case–control analysis of 5,000 samples with up to 50 heterozygotes is shown in Figure 1a while Figure 1b shows the results for 10,000 samples. A distinct pattern is seen in which the inflation factor depends on whether there are an odd or an even number of heterozygotes. This is due to the fact that there are a limited number of possible ways that small numbers of heterozygotes can be distributed between cases and controls and that for an odd number of heterozygotes the distribution between cases and controls cannot be equal. This oscillatory pattern would not be observed in analyses where the outcome is on a continuous scale for example, in time-to-event analyses, instead of a discrete scale as in a case–control analysis. The pattern observed in Figure 1 is similar to that seen in the estimation of the coverage probability of confidence intervals [18,19].

**Figure 2:**
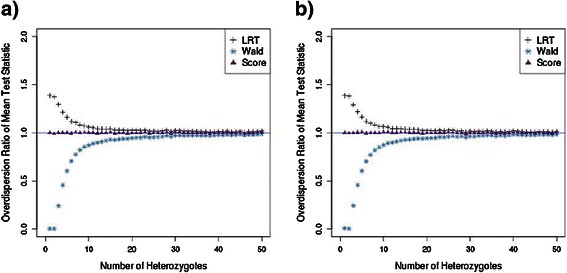
**The level of inflation in the test statistic evaluated at the mean is used to smooth out the variation in the median test statistic caused by the small number of contingencies.** We consider how the over-dispersion ratio varies as the frequency of variant increases. **a)** The over-dispersion ratio evaluated at the mean test statistic in a case–control analysis of 5,000 samples with variants with up to 50 heterozygotes. **b)** The over-dispersion ratio evaluated at the mean test statistic in a case–control analysis of 10,000 samples with variants with up to 50 heterozygotes.

The use of the mean test statistic smoothes out the variation observed in the median test statistic which was caused by the small number of contingencies. The mean LRT statistic is inflated, whereas the mean Wald test statistic is underinflated, for tests based on fewer than 20 heterozygotes (Figure 2a). The score test performs the best of the three with an inflation estimate close to 1 across a range of heterozygote frequencies. The total sample size made little difference to the pattern produced from either measure and the most important variable was the total number of heterozygotes (Figures 1b and 2b), which is dependent on allele frequency and sample size. The results from the analysis of variants of a specified allele frequency show that variants with a heterozygote frequency of less than 20 are likely to cause inflation of the test statistic.

### Determining the size of the effect of frequency on inflation factor (λ)

The proportion of the variants on the Illumina HumanExome Beadchip array that had a lower heterozygote frequency than 20 from an ovarian cancer case–control study of ~7,000 subjects was 63.2%. We therefore simulated the datasets for analysis ensuring that a representative proportion of the 100,000 variants had a heterozygote frequency of less than 20 heterozygotes.

**Figure 3:**
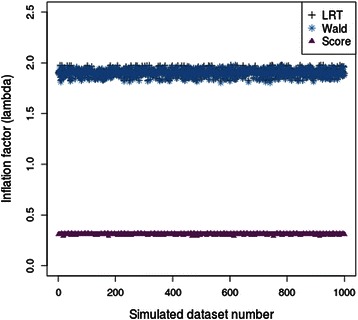
**The levels of inflation (** **λ** **) measured at the median test statistic using the likelihood ratio test, Wald test and score test on rare variant focussed datasets.** There is evidence that the test statistics of the Wald test are under-inflated whereas the test statistics for the likelihood ratio test and the score test are over-inflated.

There was substantial inflation of the test statistic for both the likelihood ratio test and the score test. In contrast, the test statistic for the Wald test was under-inflated. The values of the inflation factor (λ) for the three tests across the 1,000 simulated datasets are shown in Figure 3. This overall pattern is more pronounced when we measure the inflation of the test statistics for variants with less than 20 heterozygotes. The level of inflation of the test statistics for variants with greater than 20 heterozygotes is approximately 1 for all three tests which indicates that the properties of the tests no longer have an effect on the level of inflation in test statistics for variants with a minor allele frequency at least this great. The values of λ averaged over the 1,000 simulated datasets are presented in Table 1. The over-inflation in the LRT and score test statistics and under-inflation in the test statistics of the Wald test is consistent with the results from the datasets with variants of a single allele frequency (Figure 1). In addition, the level of inflation in the mean test statistic from the score test is approximately 1 for all groups of variants. This is consistent with the single allele frequency results in Figure 2.

**Table 1:** **The level of inflation in the mean and median test statistics of the association tests**

	**All variants**		**Variants < 20 heterozygotes**		**Variants ≥ 20 heterozygotes**	
	**λ at median**	**λ at mean**	**λ at median**	**λ at mean**	**λ at median**	**λ at mean**
LRT	1.905 (1.903-1.906)	1.172 (1.171-1.172)	3.047 (3.047-3.047)	1.270 (1.270-1.271)	0.992 (0.991-0.992)	1.002 (1.001-1.002)
Wald test	0.311 (0.310-0.311)	0.580 (0.580-0.580)	0.017 (0.017-0.017)	0.343 (0.343-0.343)	0.990 (0.989-0.991)	0.987 (0.987-0.988)
Score test	1.902 (1.900-1.904)	1.004 (1.004-1.004)	2.198 (2.198-2.198)	1.008 (1.008-1.009)	0.991 (0.990-0.992)	0.997 (0.996-0.997)

The level of inflation in the test statistics of the likelihood ratio test, Wald test and score test measured at the median test statistic and the mean statistic. The inflation factor (λ) is calculated by comparing the observed test statistic to the expected test statistic at a given point in the χ^2^ distribution. Inflation was measured for datasets including all variants, only variants with less than 20 heterozygotes in the sample and only variants with at least 20 heterozygotes in the sample. Each value of λ was averaged across 1000 simulated datasets. All intervals are 95% confidence intervals. A normal level of inflation is indicated by λ = 1.

## Conclusions

In a genetic association analysis of rare variants, the level of over-dispersion is usually evaluated at the lower end of the test statistic distribution. This method is effective in assessing bias due to population structure in analysis of common variants but if a substantial number of variants being investigated have rare allele frequencies the presence or absence of bias may be masked by the properties of the statistical test being used for analysis.

The use of either a likelihood ratio test or a score test is likely to lead to inflation of the median test statistics in the absence of population structure, if a substantial proportion of the variants have a heterozygote frequency of less than 20, independent of total sample size. On the other hand, analyses based on the Wald test may result in under-inflation of the test statistic which may mask the presence of bias due to population structure.

Thus, to ensure that the properties of the association test itself do not contribute to the assessment of bias due to population structure, the results for variants with less than 20 heterozygotes should be excluded from the calculation of λ. However, in some instances, for example, in the use of exome chip arrays, these variants make up a substantial proportion of the total number of variants.

## Additional file

Additional file 1:
**(.R) – R code for simulations.**
